# Synthesis of Silver Nano Particles Using Myricetin and the In-Vitro Assessment of Anti-Colorectal Cancer Activity: In-Silico Integration

**DOI:** 10.3390/ijms231911024

**Published:** 2022-09-20

**Authors:** Syed Tauqeer Anwer, Mohammad Mobashir, Omer I. Fantoukh, Bushra Khan, Khalid Imtiyaz, Irshad Hussain Naqvi, M. Moshahid Alam Rizvi

**Affiliations:** 1 Genome Biology Lab, Department of Biosciences, Faculty of Natural Science, Jamia Millia Islamia, New Delhi 110025, India; 2 SciLifeLab, Department of Oncology and Pathology, Karolinska Institutet, P.O. Box 1031, S-171 21 Stockholm, Sweden; 3 Department of Pharmacognosy, College of Pharmacy, King Saud University, Riyadh 11451, Saudi Arabia; 4 Ansari Health Center, Jamia Millia Islamia, New Delhi 110025, India

**Keywords:** myricetin, silver nanoparticles, colorectal cancer, in-vitro, in-silico, network-level understanding

## Abstract

The creation of novel anticancer treatments for a variety of human illnesses, including different malignancies and dangerous microbes, also potentially depends on nanoparticles including silver. Recently, it has been successful to biologically synthesize metal nanoparticles using plant extracts. The natural flavonoid 3,3′, 4′, 5,5′, and 7 hexahydroxyflavon (myricetin) has anticancer properties. There is not much known about the regulatory effects of myricetin on the possible cell fate-determination mechanisms (such as apoptosis/proliferation) in colorectal cancer. Because the majority of investigations related to the anticancer activity of myricetin have dominantly focused on the enhancement of tumor cell uncontrolled growth (i.e., apoptosis). Thus, we have decided to explore the potential myricetin interactors and the associated biological functions by using an in-silico approach. Then, we focused on the main goal of the work which involved the synthesis of silver nanoparticles and the labeling of myricetin with it. The synthesized silver nanoparticles were examined using UV-visible spectroscopy, dynamic light scattering spectroscopy, Fourier transform infrared spectroscopy, and scanning electron microscopy. In this study, we have investigated the effects of myricetin on colorectal cancer where numerous techniques were used to show myricetin’s effect on colon cancer cells. Transmission Electron Microscopy was employed to monitor morphological changes. Furthermore, we have combined the results of the colorectal cancer gene expression dataset with those of the myricetin interactors and pathways. Based on the results, we conclude that myricetin is able to efficiently kill human colorectal cancer cell lines. Since, it shares important biological roles and possible route components and this myricetin may be a promising herbal treatment for colorectal cancer as per an in-silico analysis of the TCGA dataset.

## 1. Introduction

A quick, suitable, and acceptable biosynthetic method is the phyto-mediated synthesis for the production of metal nanoparticles (NPs) [1,2,3,4]. In recent years, a variety of plant extracts, including bark, leaf, fruit, stem, and seed extracts, have been used to effectively produce metal nanoparticles. The antibacterial, antifungal, and anti-proliferative characteristics of silver (Ag) nanoparticles (NPs) have led to their widespread use among metal nanoparticles [2,5,6,7]. Due to their potent antibacterial properties, silver nanoparticles (AgNPs) have been widely used in food packaging, food and seed preservation, biofertilizers, cosmetics, and pharmaceuticals [2,8,9]. In addition to these applications, it has been discovered that silver nanoparticles are frequently employed in the areas of biomolecular detection, diagnostics, catalysis, and microelectronics [10,11,12].

Numerous physical and chemical techniques, including a reduction in solutions, chemical and photochemical reactions in reverse micelles, thermal decomposition of silver compounds, radiation-assisted, electrochemical, sonochemical, microwave-assisted processes, and, most recently, green chemistry technology, have been used for the synthesis of AgNPs. In terms of cost-effectiveness, environmental friendliness, and simplicity of scaling up for large-scale synthesis without the use of energy, high pressure, temperature, or toxic chemicals, the green synthesis approach exceeds the chemical and physical methods [3,9,13].

Studies on the creation of AgNPs from various plant sources have been reported in previous works. There is still a need for an environmentally pure, economically sound, and commercially viable method to synthesize AgNPs despite the use of new plant sources. In recent years, there has been a lot of interest in the use of plants and plant-based products in the production of different metal nanoparticles [2,6,9,10,14]. The industrial production of herbal medicine and bioactive ingredients for pharmaceutical and dietary supplements uses a range of ethnomedicinal plants. Numerous natural substances, including flavonoids, alkaloids, organic acids, volatile oils, polysaccharides, saponins, anthraquinones, terpenoids, and others, have lately shown promise in their ability to combat bacteria and viruses [2,9,12,15]. Biological activities, pharmacological effects, and antiviral properties are all shown by the family of phytochemicals known as flavonoids. A dietary flavonoid called myricetin has a wide range of biological effects, such as antibacterial, antioxidant, and anticancer characteristics. Myricetin cannot be employed in additional in-vivo experiments due to its lower bioavailability and inferior solubility in an aqueous solution. To address bioavailability concerns, several medication delivery techniques are being investigated [16,17,18,19,20,21,22].

For patients with a range of cancer types, such as melanoma, breast, and lung cancers, biomarker-driven customized therapy has made impressive progress toward precision oncology also for patients with colorectal cancer (CRC) [23,24,25,26]. Nevertheless, the availability of patient-derived CRC models along with in-vitro and in-vivo pharmacological and functional studies has led to advancements in the area during the last decade. Other variables than gene-specific mutations can affect the efficacy of targeted therapy [27,28,29,30,31,32,33]. In fact, to effectively suppress BRAF or KRAS in metastatic CRCs brought on by activating mutations in these genes, a combination of drugs is required that block the mutant protein while also limiting adaptive resistance through CRC-specific EGFR-mediated feedback loops. According to the emerging paradigm, the intrinsic biology of CRC cells must be assessed along with the molecular profiles of individual tumors in order to successfully individualize treatment [34,35,36]. As shown in the previous work, the design of practice-changing clinical trials was supported by preclinical investigations based on patient-derived models. The fusion of diverse experiences into a unified framework will transform the design of biology-informed clinical trials in this field in the future [32,37,38,39,40].

Cancer develops and spreads as a result of the accumulation of biological traits that may help tumor cells thrive, proliferate, escape immune monitoring, and become more resilient under challenging circumstances. The creation of tumor molecular maps has aided in the development of novel inhibitors that specifically target the altered genes and signaling pathways driving the malignant phenotype [38,41,42,43,44]. Therefore, molecular biomarkers should be used to direct the creation of efficiently targeted medications and to individually tailor treatments for each patient. However, it has been challenging to apply the same logic to CRC due to the genetic heterogeneity of this specific cancer type and the lack of druggable targets. The numerous molecular alterations that have been described in CRC throughout the years have led to the evolution of a few essential approaches for choosing and validating actionable targets [40,45,46,47,48].

The second deadliest and third most common disease in the world and based on the available reports, CRC is known to cause more than 1.7 million new cases and approximately 862,000 deaths in 2018 [49]. Treatment options for colorectal cancer range from surgery to radiation therapy to chemotherapy to targeted treatments such as inhibiting the checkpoint and angiogenesis. Some individuals who get traditional chemotherapeutics acquire drug resistance despite better therapy options. New chemotherapeutic drugs are thus needed for clinical practice [27,28,29,50,51].

Numerous foods, such as fruits, herbs, and nuts, contain a pigment known as myricetin (3,3′,4′,5,5′,7-hexahydroxyflavone), which is a kind of flavonoid. The 3′,4′,5′-trihydroxy group on myricetin makes it unique from the other flavonols. It has been demonstrated that myricetin has anticancer properties in a number of cancers, including prostate, breast, stomach, and CRCs [2,16,17,18,20,52]. There haven’t been many studies, nevertheless, on myricetin’s role in colorectal cancer. It has been reported that myricetin suppressed the development of colorectal cancer both in-vitro and in-vivo. Myricetin causes apoptosis in HCT-115 colon cancer cells via raising the expression of nucleoside diphosphate kinase and other proteins that are controlled by a caspase [52,53]. A flavonol called myricetin is present in many foods, such as berries, herbs, and walnuts. Previous studies have demonstrated that myricetin has anticancer capabilities against a number of malignancies, including pancreatic, cutaneous, and hepatocellular tumors [52]. As mentioned above that a number of nanoparticles are used in combination with herbal drugs also against cancers. Pure myricitin is also known as having anticancer effects, so we decided to evaluate pure myricetin and myricetin coated with AgNPs against one of the leading cancer types, CRC.

## 2. Results

**Figure 1:**
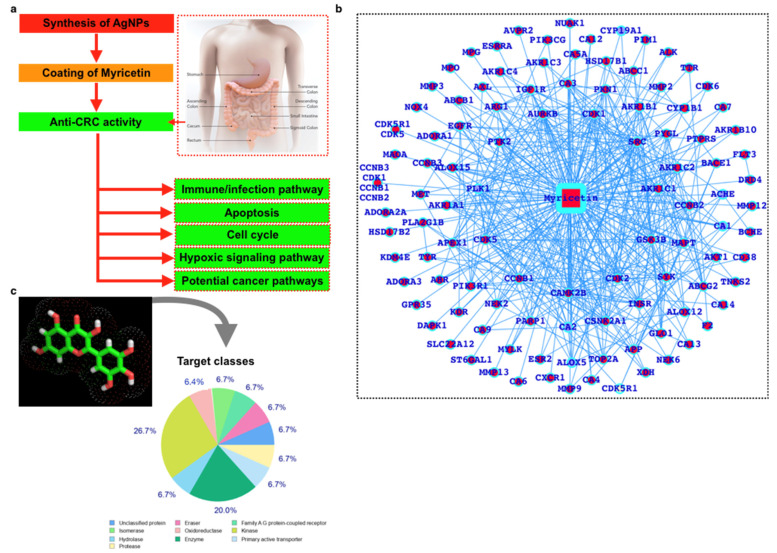
(**a**) Workflow, (**b**) myricetin interactors, and (**c**) the panther classes of the interactors.

Because conventional cancer medicines have intrinsic limits, many nanotechnologies have been developed and put to use to treat cancer more effectively and safely, a field known as cancer nanomedicine. Although there has been significant technological advancement in this area, the complexity and heterogeneity of tumor biology, a lack of knowledge of nano-bio interactions, and the difficulties in chemistry, manufacturing, and control that must be overcome for clinical translation and commercialization are the main obstacles to nanomedicine becoming a new paradigm in cancer therapy. Here, we have used a novel strategy to demonstrate the increased efficacy of nano-therapeutics against cancer patients by utilizing our knowledge of antitumor and nano-bio interactions. The summarized steps have been presented in Figure 1a. Myricetin interacts with a number of potential proteins associated with cancer including colorectal cancer: As an initial step, we performed the screening of the putative interactors (proteins) (Figure 1b) and performed the protein classification (Figure 1c). The results show a large number of proteins could be the putative targets for myricetin. In KEGG pathway analysis, we observe that there are critical pathways that are well-known to be directly regulating CRC and these pathways are colorectal cancer pathway, pathways in cancer, endometrial cancer, prostate cancer, basal cell carcinoma, melanogenesis, bladder cancer, pancreatic cancer, non-small cell lung cancer, small cell lung cancer, glioma, acute myeloid leukemia, cell cycle, Wnt signaling, ErbB signaling, p53 signaling, cytokine and chemokine signaling, VEGF signaling, JAK-STAT signaling, apoptosis, and the major immune signaling pathways (Supplementary Table S1). There were 129 pathways in total which were associated with the myricetin target proteins for the 82 proteins which were the target proteins. Some of these 82 proteins associate more with a number of pathways while some were associated with a few pathways. CAMK2B, GSK3B, PIK3CG, MET, IGF1R, AKT1, PIK3R1, EGFR, PTK2, and PLA2G1B were associated with more number of pathways which means these proteins could affect more biological functions.

### 2.1. AgNPs Synthesis and Characterization

**Figure 2:**
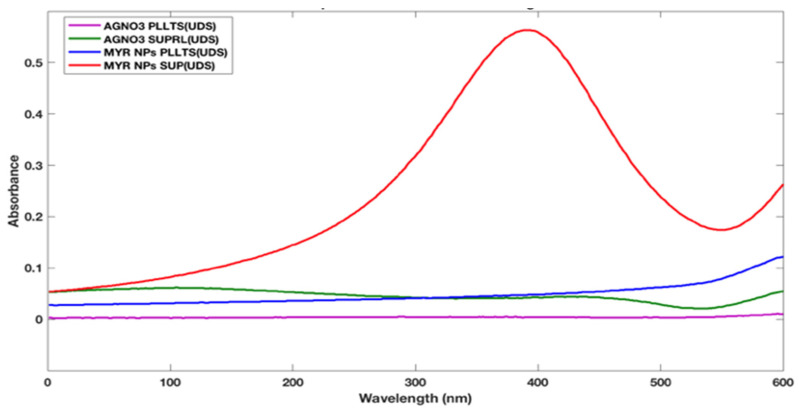
UV-vis spectra of Myricetin and the AgNPs.

As mentioned in the previous section the significance of myricetin and its potential role in cancer mainly CRC, thus we proceeded to our next goal where we wanted to label this herbal drug with AgNPs so we proceeded towards the synthesis and characterization as an initial step. The UV-vis spectra has been used for the investigation of proper embedding/labeling of myricetin with AgNPs and the UV-vis spectra of myricetin extract and the AgNPs were displayed in Figure 2. The different colors were for different sets of data. These spectra were from the UV-vis spectra. The absorption spectra of the extract are in the range of 300–500 nm while AgNPs formed by both the methods have a peak wavelength of 380 nm. These alterations are the result of AgNPs’ surface plasmon resonance rapidly changing. The widening of the peak, which implies the creation of polydispersed big nanoparticles as a result of sluggish reduction rates, reveals this alteration. AgNPs-mw had a greater maximum absorbance than AgNP-aging, which suggests that bioreduction was accomplished more quickly. In comparison to Ag NPs-aging, Ag NPs-mw has a darker final color. The peak is observed at 380 nm which leads to the conclusion that Myricetin has been successfully labeled by AgNPs.

The presence of nanoparticles was confirmed by obtaining a spectrum in the visible range of 300 nm to 500 nm using a UV-visible spectrophotometer (Figure 2). From this analysis, absorbance peak was found at around 420 nm, which was specific for Ag nano-particles. Based on the UVeVIS spectra, the sharpness of the absorption peak was found to be dependent on the concentration ratio of the leaf extract; thus, it was sharper with a higher concentration ratio (Figure 2). A previous study had also been the effect of varying the concentration of leaf extract on the size of AgNPs. The results obtained by them were very similar to the data we have presented. As the concentration of leaf extract is increased, a greater number of biomolecules is available in the leaf extract which will be helpful for the metal reductive process.

Aqueous solution (1 mM) of silver nitrate (AgNO_3_) was prepared in 250 mL conical flasks, and myricetin (1 mM) was added for reduction into Ag^+^ ions. The mixture was then stirred overnight. By using UV-vis spectrophotometric analysis, the color shift of myricetin from light yellowish to reddish brown was noted. The identical combination was made and aged for 24 h prior to being observed using UV-vis spectrophotometry as a comparison. The solution was filtered to produce tiny particles so that they could be thinly coated on the glass surface for XRD and SEM investigation. AgNPs-mw and AgNPs-aging are the names given to the AgNPs produced by microwave irradiation and aging processes, respectively. The volume ratio in the AgNPs-mw preparation was adjusted at a 10:1 ratio in order to assess the impact of the silver nitrate solution’s volume ratio relative to the myricetin on the particle size distribution and its antibacterial activity.

### 2.2. Morphological Characteristics of BioAgNPs

**Figure 3:**
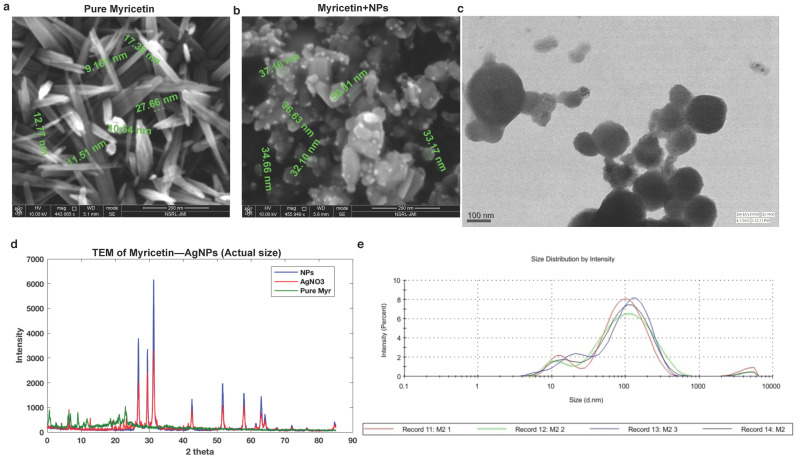
(**a**) Scanning electron microscope (SEM) of pure Myricetin, (**b**) SEM of Myricetin coated with NPs (AgNPs), (**c**,**e**) TEM of Myricetin—AgNPs, (**d**) comparative XRDs of NPs, AgNO_3_, and Myricetin, and (**e**) particle diameter analysis for AgNPs (dynamic light scattering (DLS) analysis).

The microstructure of the BioAgNPs or mAgNPs (myricetin silver nano-particles) synthesized and was analyzed by scanning electron microscopy (SEM). Figure 3a,b show SEM profiles of the drop-coated AgNPs films under various magnifications. The photos show the aggregate AgNPs creation using both approaches, which have a spherical-like structure. The sample preparation process for the sample, which requires the nanoparticles to be filtered and dried before measurement, is connected to the aggregate formation in SEM analysis. Particles with different surface morphologies were discovered using a variety of techniques; for example, microwave-assisted AgNPs produce flakes, whereas aging produces aggregates that resemble spheres.

The produced AgNPs are in the 12–20 nm nanoparticle size range, according to TEM profiles in Figure 3c. The TEM pictures display a variety of morphologies, with spherical shapes predominating. A thin layer of plant-derived organic material can also be seen in the photos and has been mentioned in several earlier studies that used plant extracts.

With the use of Shimadzu X-6000 (Kyoto, Japan) equipment and Rietica, the Rietveld refinement of AgNPs was carried out. Before analysis, the powdered AgNPs were spread over a glass film after the sample’s solvent had evaporated (Figure 3d).

XRD is an analytical technique used to identify the phase of a crystalline substance and to study the structure of crystals. In this technique, X-rays are produced in a cathode ray tube and get filtered to form monochromatic radiation that interacts with the sample and forms constructive interference. AgNPs synthesized using the Myricetin confirm the XRD pattern, as shown in Figure 3c in which the diffraction peaks are in the 2θ ranges which were consistent with the structure of Ag. The size of biosynthesized AgNPs was calculated using Debye–scherrer equation (Equation (1)).

(1) $$D=\frac{0.94\lambda }{\beta \cos\theta }$$

where D is the mean particle size, λ is the X-ray wavelength, β is the line broadening at half the maximum intensity and θ is the Bragg angle. The result was in close agreement with the result of the TEM measurement. The crystallite size is calculated to be approximately 18 nm and 17 nm. The Ag NPs-mw are slightly bigger than Ag NPs-aging. Furthermore, we have also presented particle diameter analysis for AgNPs (dynamic light scattering (DLS) analysis) which confirms that the synthesized nanoparticles were AgNPs (average diameter was approximately 61 nm) (Figure 3e).

The distribution implies that during the fast reduction process brought on by microwave irradiation, particle aggregates are formed with increased energy transfer. The shift in the volume ratio suggests that the particle size diameter distribution of the AgNPs will be smaller the greater the concentration of Myricetin extract. The means of the particle size distribution are displayed in Figure 3a,b.

**Figure 4:**
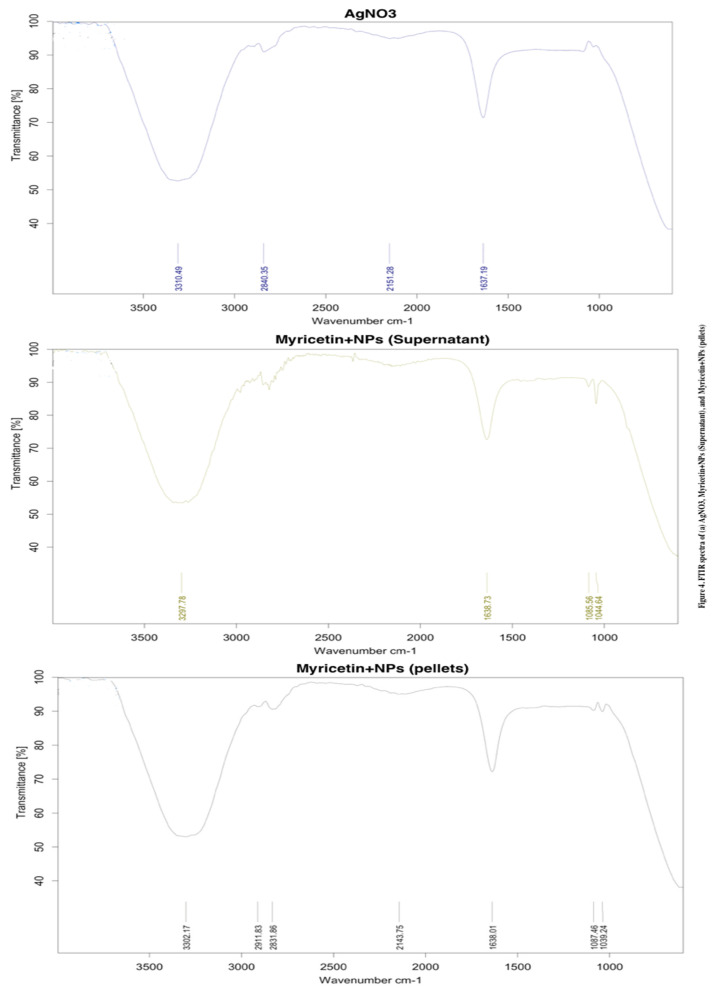
FTIR spectra for myricetin with and without NPs.

A comparison of the FTIR spectra of the BP extract and AgNPs is displayed in Figure 4. The FT-IR spectra of BP extract show several major peaks at 3292, 2917, 2849, 2112, 1742, 1630, 1420, 1375, 1147, and 1043 cm^−1,^ and some other peaks approximately at 1000 cm^−1^. The peak at 3292 cm^−1^ represents the AOH stretching vibration from phenolic compounds in the extract while the three peaks at 2849, 1375, and 1043 cm^−1^ are probably attributable to the NH stretching vibration of amide II, CAO stretch, and CAN stretching of amines, respectively. The peak at 1630 cm^−1^ is designated as the stretching vibration of the C‚O bond. After the Ag NPs formation, there are some shifts of valuable peaks such as the O-H vibration from 3292 to 3306 cm^−1^, C‚O vibration from 1630 to 1634 cm^−1^, and NAH vibration from 1043 to 1045 cm^−1^, indicating that reduction occurred. Overall, the spectra of AgNPs-mw and Ag NPs-aging are similar. FTIR data suggest that its functional group (-COOH) has been identified.

### 2.3. Human Colorectal Cancer Cells’ Vitality Was Decreased by Myricetin

**Figure 5:**
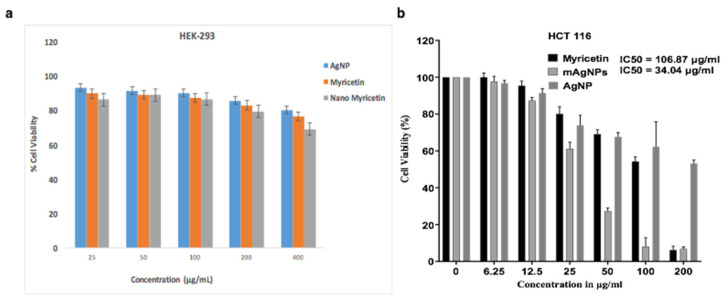
MTT assay (anti-CRC activity). MTT assay was performed to investigate the (**a**) effect of pure myricetin, AgNPs, and mAgNPs (myricetin labeled with AgNPs) in normal cell line HEK293 and (**b**) the anti-CRC activity of pure myricetin, AgNPs, and mAgNPs AgNPs for CRC cell line HCT-116.

Using cell viability assay, we evaluated the effects of AgNPs, pure myricetin, and myricetin labeled with AgNPs (mAgNPs) in normal cell line i.e., control (HEK-293) (Figure 5a) and in a human colorectal cancer cell line (HCT116) (Figure 5b). For 48 h, colorectal cells were exposed to different doses of mAgNPs and myricetin (6.25, 12.5, 25, 50, 100, and 200 µg/mL). HCT116 cells were shown to be more highly sensitive to myricetin than mAgNPs, with IC50 values of 106.87 μg/mL and 34.04 µg/mL, respectively (Figure 5). As a result, this cell line was chosen for further research. In a dose-dependent way, myricetin drastically reduced the growth of HCT116 cells. AgNPs showed biocompatibility when treated with normal cell line HEK-293. The nanoformulation does not show enough cytotoxicity up to 400 μg/mL concentration while in the case of cancer cell line it causes 50 percent death below 200 μg/mL concentration. In the case of normal cell line HEK-293, there was not much significant effect as shown in Figure 5a.

### 2.4. CRC Gene Expression Profiling and Functional Enrichment Analysis

**Figure 6:**
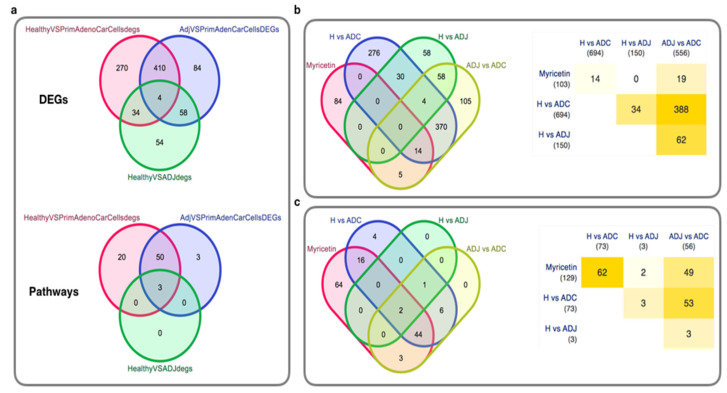
CRC gene expression profiling and pathway enrichment analysis. (**a**) Venn diagram to show the specific and shared DEGs and enriched pathways in the case of CRC with different types of samples. (**b**) Venn diagram to show the specific and shared DEGs and common DEGs with myricetin targets. (**c**) Pathway-level linkage of myricetin and the CRC. The degree of yellow color in (**b**) and (**c**) represents the less (low degree) to higher (higher degree) number of overlapping genes.

Finally, we have selected the CRC dataset (GSE44076) with gene expression and performed gene expression and pathway enrichment analysis. The selected dataset contains the healthy, adjacent, and primary adenocarcinoma samples, so we have compared healthy with adjacent samples, healthy with primary adenocarcinoma, and adjacent with primary adenocarcinoma samples and thus generating three differentially expressed genes (DEGs) list (Figure 6a). Further, the three DEGs lists were processed for pathway enrichment analysis (Figure 6a). In the CRC gene expression profiling, we observed that there were four genes only which were common to all (healthy versus primary adenocarcinoma cells DEGs list, adjacent cells versus primary adenocarcinoma cells DEGs list, and healthy versus adjacent cells DEGs list), 410 common between healthy versus primary adenocarcinoma cells DEGs list and adjacent cells versus primary adenocarcinoma cells DEGs list, 34 genes were common between healthy versus primary adenocarcinoma cells DEGs list and healthy versus adjacent cells DEGs list, and 58 genes were common between healthy versus adjacent cells DEGs list and adjacent cells versus primary adenocarcinoma cells DEGs list. While 270 genes were specific to healthy versus primary adenocarcinoma cells DEGs list, 54 genes were specific to healthy versus adjacent cells DEGs list, and 84 genes were specific to adjacent cells versus primary Adenocarcinoma cells DEGs list.

In pathway enrichment analysis, we observe that there were only three pathways that were common to all (healthy versus primary adenocarcinoma cells DEGs list, adjacent cells versus primary adenocarcinoma cells DEGs list, and healthy versus adjacent cells DEGs list), 50 common between healthy versus primary adenocarcinoma cells DEGs list and adjacent cells versus primary adenocarcinoma cells DEGs list, no pathway was common between Healthy versus primary adenocarcinoma cells DEGs list and healthy versus adjacent cells DEGs list, and no pathway was common between healthy versus adjacent cells DEGs list and adjacent cells versus primary adenocarcinoma cells DEGs list (Table 1). While 20 pathways were specific to healthy versus primary adenocarcinoma cells DEGs list, no pathway was specific to healthy versus adjacent cells DEGs list, and three pathways were specific to adjacent cells versus primary adenocarcinoma cells DEGs list. ADH1B, COL1A2, MEP1B, and MGP genes and PI3K-Akt signaling, focal adhesion, and ECM-receptor interaction pathways were commonly differentially expressed and enriched for all the three DEGs lists, respectively. Furthermore, we have also compared the Myricetin interactors and the inferred pathways with the CRC gene expression analysis outcome for DEGs and enriched pathways (Figure 6b,c). Here, we clearly see that myricetin interactors and pathways are also shared with CRC. Myricetin shares 14 genes with healthy versus adjacent cells DEGs list and 19 genes with adjacent cells versus primary adenocarcinoma cells DEGs list and in case of pathways, myricetin shares 62 pathways with healthy versus adjacent cells DEGs list, two with healthy versus adjacent cells DEGs list, and 49 with adjacent cells versus primary adenocarcinoma cells DEGs list. This result suggests that myricetin could be the potential herbal drug to target the CRC pathways and pathway components (genes) (Figure 6b,c). Among these highly enriched pathways, the majority of them directly associated with cancer and also the CRC and also similar to the individual DEGs lists also have genes, majority of whom could be either oncogenes or the genes which are potentially responsible for the alterations of cancer associated biological functions.

## 3. Discussion

Chinese herbal therapy has been found to enhance immune function, encourage reticuloendothelial system phagocytosis, safeguard the bone marrow’s ability to produce blood cells, and prevent drops in platelets and white blood cells. Traditional Chinese herbal remedies have become more and more popular in recent years for the adjuvant treatment of cancer [21,53,54,55,56]. A natural flavonoid called myricetin is extensively distributed in many different types of plants, including the leaves of *Ampelopsis sinica* (Miq.) W.T. Wang and *Camellia sinensis* (L.) Kuntze and the bark of *Myrica nagi* Thunb [16,22,57]. The majority of research on myricetin’s antitumor mechanism has been on how it affects apoptosis and inflammation, suggesting a potential involvement in the development of cell growth. The effects of myricetin on the induction of colon cancer cell apoptosis and autophagy were examined in the current investigation. The anti-colon tumor effects of myricetin were brought on via apoptotic and autophagic mechanisms [2,16,53,58,59].

A crucial intracellular signaling mechanism for controlling the cell cycle is the PI3K/AKT/mTOR pathway. As a result, it has a direct bearing on cellular dormancy, proliferation, cancer, and aging. According to Phillips PA, myricetin triggered apoptosis and inhibited the PI3K signaling pathway, which led to the death of pancreatic cancer cells. The PI3K/AKT/mTOR signaling pathway may control autophagy and have an impact on cellular growth. Western blotting was performed in our work to identify the various signaling pathways in myricetin-treated HCT116 and SW620 cells, and the results showed that the PI3K/AKT/mTOR signaling pathway was responsible for inducing cell death and autophagy.

The inhibitory effects of myricetin on colon cancer entail both apoptosis and autophagy, two different types of cell death. Previous studies have discovered that the processes of autophagy and apoptosis take place in the same cells [35,36]. According to reports, mTOR and AMPK signaling connect autophagy with apoptosis. Numerous studies have indicated autophagy-induced apoptosis and the activation of the AMPK and Bax signaling pathways. Nevertheless, several studies in recent years have demonstrated that blocking autophagy might make cancer cells more susceptible to chemotherapeutic treatments by lowering drug resistance. The development of cancer is thought to involve autophagy in two different ways. Autophagy slows the development of cancer cells in the early stages of the disease. However, as a defense mechanism in advanced cancer, autophagy can also aid in the growth of tumors. In our research, we discovered that myricetin accelerated the apoptosis of human colorectal cancer cells by suppressing autophagy with 3-MA. Therefore, myricetin-induced apoptosis of colorectal cancer cells was increased by autophagy suppression. A crucial mechanism through which colon cancer cells gain myricetin resistance is the protective impact of autophagy. The combination of myricetin with autophagy inhibitors may be an effective alternative for chemotherapy or adjuvant chemotherapy since myricetin alone suppresses colon cancer cell growth and promotes apoptosis. Future research on the mechanism of myricetin is still required. The discovery and implementation of different nanotechnologies for more efficient and safe cancer treatment—hereafter referred to as cancer nanomedicine—was spurred by the inherent limitations of conventional cancer treatments.

## 4. Materials and Methods

### 4.1. Collection and Preparation of Plant Extract (Myricetin)

We have directly collected the herbal drug myricetin (M670-25MG) from Sigma-Aldrich which has CAS 529-44-2.

### 4.2. Biosynthesis of Silver Nanoparticles (AgNPs)

The appropriate reaction mixture for the biogenesis of silver nanoparticles was created by mixing 1 mL of myricetin with 9 mL of 1 mM AgNO_3_ solution; the AgNO_3_ was purchased from MERCK. As a control, however, the identical experimental setup with 1 mL of myricetin and 9 mL of distilled water was maintained. Both flasks were incubated at 25 °C in the dark for 2–4 h using a rotary shaker. Later, the generated AgNPs were separated and purified by continuous centrifugation in sterile miliQ water at 10,000 rpm for 20 min at 4 °C. In order to further characterize and examine the bioactivity of the dried AgNPs, they were stored at 4 °C [4,6,8].

### 4.3. Characterization of Silver Nanoparticles

The biosynthesis of the AgNPs (bio reduction of the Ag^+^ ions) in an aqueous solution was monitored periodically in a UV-Vis spectrophotometer (Hitachi U3900) within the range of 200–600 nm. The UV-visible spectra of the resulting reaction solution were monitored as a function of reaction time at a resolution of 1nm room temperature (25 °C). The purified samples were 10-folds diluted with the phosphate buffer saline PBS (0.15 M, pH 7.4). The aliquots were later sampled in dynamic light scattering (DLS) cuvettes and examined for equivalent diameters, size distribution, and zeta potential. The particle diameters were assessed at a scattering angle of 90° at room temperature (25 °C). Fourier Transform Infra-Red spectra of the AgNPs were studied in an FT-IR spectrophotometer (Bruker Tensor 37) in transmission (%) mode with 200 scans. The AgNPs were pelletized with potassium bromide (KBr) having a 1% sample concentration (*w*/*w*) and were analyzed against the background of a pure KBr pellet. The nano-scale size of silver particles was confirmed by the analysis of morphological structure under a scanning electron microscope (Sigma BP, Zeiss, Germany) performed at an acceleration voltage of 15 KV [4,10,60].

### 4.4. Cell Culture and Treatment

From NCCS Pune in India, HCT116 was gathered. The cells were grown in DMEM media with 10% foetal bovine serum (FBS) at 37 °C in a 5% CO_2_ environment [61,62,63].

### 4.5. Cell Viability Assay

The MTT cell viability test was used to gauge cell viability. In 96-well culture plates, the cells were planted at a density of 1 × 104 cells/well. Different myricetin concentrations (0, 12.5, 25, 50, 100, and 200 mol/L) were added after 4 h, and the mixture was then incubated for 24 h. As a control, cells were treated in culture media containing a similar volume of dimethyl sulfoxide (DMSO). To calculate the cell survival rate, the absorbance was measured using a microplate reader at 570 nm excitation/emission wavelengths [64,65].

### 4.6. Transmission Electron Microscopy

With the help of traditional electron microscopy, cell size was determined. Prior to being treated with 1 percent OsO_4_ for 1 h, the cells were first fixed for 2 h with 2.5 percent glutaraldehyde and 2.5 percent formaldehyde. The cells were then treated progressively with acetone/resin Spur’s (1,1), ethanol, and 100 percent Spur’s resin after being soaked in 2 percent uranyl acetate for 1 h. The ultrathin cell sections were seen using transmission electron microscopy [2,64,66].

### 4.7. Statistical Analysis

*t*-test statistical analysis was conducted using SPSS/Win 13.0 program for pair samples. The findings are shown as the mean ± SD, and significant findings are denoted by the following symbols: * *p* = 0.05, ** *p* = 0.01 and *** *p* = 0.001.

### 4.8. Gene Expression and Pathway Enrichment Analysis

In this study, we proposed to investigate the genes and signaling pathways that are active in lung cancer patients, as well as to find potential targets and herbal remedies that might interfere with these processes [67,68,69,70]. To be more precise, we employed an integrated strategy to identify the up- or down-regulated genes of particular cancer patients before searching for potential transcription factors that had been activated.

In the first step, we have selected the data of our interest (raw expression datasets) GSE44076 (https://www.ncbi.nlm.nih.gov/geo/query/acc.cgi?acc=GSE44076) dataset contains healthy, adjacent, and adenocarcinoma samples and processed it until normalization and log2 values of all the mapped out the genes as mentioned.

We have compared the samples (control) to the corresponding samples (target/CRC) for differential gene expression analysis, yielding DEGs lists. Raw file processing, intensity computation, and normalization are the basic procedures involved in the entire study. GCRMA, RMA, and EB are the most often used techniques for normalization. For raw intensity normalization, EB was utilized for raw intensity normalization. Following normalization, we moved toward the main goal of understanding gene expression patterns [44,67] and their inferred functions [67,71].

In order to generate DEGs lists, we compared three distinct types of samples to their corresponding samples for differential gene expression analysis. The three main processes that are used during the whole study are raw file processing, intensity computation, and normalization. The three methods for normalizing that are most frequently used are GCRMA, RMA, and EB [72,73,74,75,76]. We have used EB to normalize the raw intensity [77,78,79]. Normalization is followed by our major objective, which is to comprehend gene expression patterns and their presumed roles [44,67,71].

The mattes MATLAB function runs a two-sample *t*-test to analyze the differential expression of genes from two experimental circumstances or phenotypes. MATLAB tools, such as mattest, have been utilized for differential gene expression prediction and statistical analysis. In this situation, a matrix of gene expression levels is required, with each row denoting a gene and each column denoting a replication. Target and normal data both need to have the same number of rows and be normally distributed with comparable variations across classes. We have used the KEGG [80] database for pathway research and mapped out the pathways and the network analysis [81,82,83,84,85,86,87,88,89]. FunCoup2.0 [90] was used to generate all of the DEGs networks used in this study, and cytoscape [91] was used to visualize the networks. MATLAB has been used for the majority of our code and calculations.

## 5. Conclusions

Numerous malignancies have shown myricetin to have anticancer effects. The precise processes behind these impacts are not entirely understood, yet. There is limited research on myricetin’s effectiveness as a colon cancer anticancer agent. Based on our results we conclude that, myricetin drastically reduced the growth of HCT116 cells in a dose-dependent way and after 100 µg/mL, the cytotoxicity appears to be stable. Furthermore, myricetin interactors and pathways are also shared with CRC via critical biological functions and its components. This result suggests that myricetin could be the potential herbal drug to target the CRC pathways and pathway components. This study could add to the body of data supporting the use of myricetin as a colon cancer therapy. The findings form the basis for creating natural product-based anti-colon cancer medications that are more effective. Future studies are required to provide highly efficient antitumor medications that specifically target cancer cells.

## Figures and Tables

**Table 1:** Enriched pathways analysis by using Venn diagram.

Pathway	Present in
KEGG_04151_PI3K-Akt_signaling	Healthy versus Primary Adenocarcinoma, Adjacent cells versus Primary Adenocarcinoma, Healthy versus Adjacent cells
KEGG_04510_Focal_adhesion	Healthy versus Primary Adenocarcinoma, Adjacent cells versus Primary Adenocarcinoma, Healthy versus Adjacent cells
KEGG_04512_ECM-receptor_interaction	Healthy versus Primary Adenocarcinoma, Adjacent cells versus Primary Adenocarcinoma, Healthy versus Adjacent cells
KEGG_00040_Pentose_and_glucuronate_interconversions	Healthy versus Primary Adenocarcinoma, Adjacent cells versus Primary Adenocarcinoma
KEGG_00071_Fatty_acid_metabolism	Healthy versus Primary Adenocarcinoma, Adjacent cells versus Primary Adenocarcinoma
KEGG_00120_Primary_bile_acid_biosynthesis	Healthy versus Primary Adenocarcinoma, Adjacent cells versus Primary Adenocarcinoma
KEGG_00140_Steroid_hormone_biosynthesis	Healthy versus Primary Adenocarcinoma, Adjacent cells versus Primary Adenocarcinoma
KEGG_00230_Purine_metabolism	Healthy versus Primary Adenocarcinoma, Adjacent cells versus Primary Adenocarcinoma
KEGG_00260_Glycine__serine_and_threonine_metabolism	Healthy versus Primary Adenocarcinoma, Adjacent cells versus Primary Adenocarcinoma
KEGG_00350_Tyrosine_metabolism	Healthy versus Primary Adenocarcinoma, Adjacent cells versus Primary Adenocarcinoma
KEGG_00500_Starch_and_sucrose_metabolism	Healthy versus Primary Adenocarcinoma, Adjacent cells versus Primary Adenocarcinoma
KEGG_00561_Glycerolipid_metabolism	Healthy versus Primary Adenocarcinoma, Adjacent cells versus Primary Adenocarcinoma
KEGG_00564_Glycerophospholipid_metabolism	Healthy versus Primary Adenocarcinoma, Adjacent cells versus Primary Adenocarcinoma
KEGG_00590_Arachidonic_acid_metabolism	Healthy versus Primary Adenocarcinoma, Adjacent cells versus Primary Adenocarcinoma
KEGG_00830_Retinol_metabolism	Healthy versus Primary Adenocarcinoma, Adjacent cells versus Primary Adenocarcinoma
KEGG_00860_Porphyrin_and_chlorophyll_metabolism	Healthy versus Primary Adenocarcinoma, Adjacent cells versus Primary Adenocarcinoma
KEGG_00910_Nitrogen_metabolism	Healthy versus Primary Adenocarcinoma, Adjacent cells versus Primary Adenocarcinoma
KEGG_00980_Metabolism_of_xenobiotics_by_cytochrome_P450	Healthy versus Primary Adenocarcinoma, Adjacent cells versus Primary Adenocarcinoma
KEGG_00982_Drug_metabolism_-_cytochrome_P450	Healthy versus Primary Adenocarcinoma, Adjacent cells versus Primary Adenocarcinoma
KEGG_00983_Drug_metabolism_-_other_enzymes	Healthy versus Primary Adenocarcinoma, Adjacent cells versus Primary Adenocarcinoma
KEGG_03320_PPAR_signaling_pathway	Healthy versus Primary Adenocarcinoma, Adjacent cells versus Primary Adenocarcinoma
KEGG_04010_MAPK_signaling_pathway	Healthy versus Primary Adenocarcinoma, Adjacent cells versus Primary Adenocarcinoma
KEGG_04014_Ras_signaling	Healthy versus Primary Adenocarcinoma, Adjacent cells versus Primary Adenocarcinoma
KEGG_04015_Rap1_signaling	Healthy versus Primary Adenocarcinoma, Adjacent cells versus Primary Adenocarcinoma
KEGG_04020_Calcium_signaling_pathway	Healthy versus Primary Adenocarcinoma, Adjacent cells versus Primary Adenocarcinoma
KEGG_04022_cGMP-PKG_signaling	Healthy versus Primary Adenocarcinoma, Adjacent cells versus Primary Adenocarcinoma
KEGG_04024_cAMP_signaling	Healthy versus Primary Adenocarcinoma, Adjacent cells versus Primary Adenocarcinoma
KEGG_04060_Cytokine-cytokine_receptor_interaction	Healthy versus Primary Adenocarcinoma, Adjacent cells versus Primary Adenocarcinoma
KEGG_04064_NF-kappa_B_signaling	Healthy versus Primary Adenocarcinoma, Adjacent cells versus Primary Adenocarcinoma
KEGG_04068_FoxO_signaling	Healthy versus Primary Adenocarcinoma, Adjacent cells versus Primary Adenocarcinoma
KEGG_04080_Neuroactive_ligand-receptor_interaction	Healthy versus Primary Adenocarcinoma, Adjacent cells versus Primary Adenocarcinoma
KEGG_04110_Cell_cycle	Healthy versus Primary Adenocarcinoma, Adjacent cells versus Primary Adenocarcinoma
KEGG_04152_AMPK_signaling	Healthy versus Primary Adenocarcinoma, Adjacent cells versus Primary Adenocarcinoma
KEGG_04270_Vascular_smooth_muscle_contraction	Healthy versus Primary Adenocarcinoma, Adjacent cells versus Primary Adenocarcinoma
KEGG_04310_Wnt_signaling_pathway	Healthy versus Primary Adenocarcinoma, Adjacent cells versus Primary Adenocarcinoma
KEGG_04350_TGF-beta_signaling_pathway	Healthy versus Primary Adenocarcinoma, Adjacent cells versus Primary Adenocarcinoma
KEGG_04360_Axon_guidance	Healthy versus Primary Adenocarcinoma, Adjacent cells versus Primary Adenocarcinoma
KEGG_04371_Apelin_signaling	Healthy versus Primary Adenocarcinoma, Adjacent cells versus Primary Adenocarcinoma
KEGG_04392_Hippo_Signaling_Pathway	Healthy versus Primary Adenocarcinoma, Adjacent cells versus Primary Adenocarcinoma
KEGG_04514_Cell_adhesion_molecules_(CAMs)	Healthy versus Primary Adenocarcinoma, Adjacent cells versus Primary Adenocarcinoma
KEGG_04530_Tight_junction	Healthy versus Primary Adenocarcinoma, Adjacent cells versus Primary Adenocarcinoma
KEGG_04550_Signaling_pathways_regulating_pluripotency_of_stem_cells	Healthy versus Primary Adenocarcinoma, Adjacent cells versus Primary Adenocarcinoma
KEGG_04610_Complement_and_coagulation_cascades	Healthy versus Primary Adenocarcinoma, Adjacent cells versus Primary Adenocarcinoma
KEGG_04611_Platelet_activation	Healthy versus Primary Adenocarcinoma, Adjacent cells versus Primary Adenocarcinoma
KEGG_04630_Jak-STAT_signaling_pathway	Healthy versus Primary Adenocarcinoma, Adjacent cells versus Primary Adenocarcinoma
KEGG_04640_Hematopoietic_cell_lineage	Healthy versus Primary Adenocarcinoma, Adjacent cells versus Primary Adenocarcinoma
KEGG_04668_TNF_signaling	Healthy versus Primary Adenocarcinoma, Adjacent cells versus Primary Adenocarcinoma
KEGG_04670_Leukocyte_transendothelial_migration	Healthy versus Primary Adenocarcinoma, Adjacent cells versus Primary Adenocarcinoma
KEGG_04810_Regulation_of_actin_cytoskeleton	Healthy versus Primary Adenocarcinoma, Adjacent cells versus Primary Adenocarcinoma
KEGG_04916_Melanogenesis	Healthy versus Primary Adenocarcinoma, Adjacent cells versus Primary Adenocarcinoma
KEGG_04919_Thyroid_hormone_signaling_pathway	Healthy versus Primary Adenocarcinoma, Adjacent cells versus Primary Adenocarcinoma
KEGG_04920_Adipocytokine_signaling_pathway	Healthy versus Primary Adenocarcinoma, Adjacent cells versus Primary Adenocarcinoma
KEGG_04924_Renin_secretion	Healthy versus Primary Adenocarcinoma, Adjacent cells versus Primary Adenocarcinoma

## Data Availability

Not applicable.

## Supplementary Materials

The following supporting information can be downloaded at: https://www.mdpi.com/article/10.3390/ijms231911024/s1.
