# Supplementary material for: Synthesis of Silver Nano Particles Using Myricetin and the In-Vitro Assessment of Anti-Colorectal Cancer Activity: In-Silico Integration
Source: Int J Mol Sci. 2022 Sep 20;23(19):11024. doi: 10.3390/ijms231911024 (PMC9570303; doi:10.3390/ijms231911024)
Supplement: Supplementary file 1 [file ijms-23-11024-s001.zip › ijms-1879883-supplementary.pdf]

Table S1: Kegg pathways inferred for Myricetin interactors.

Protein	Pathways
CD38	KEGG_00760_Nicotinate_and_nicotinamide_metabolism
CD38	KEGG_04020_Calcium_signaling_pathway
ALOX5	KEGG_00590_Arachidonic_acid_metabolism
ALOX5	KEGG_00591_Linoleic_acid_metabolism
CAMK2B	KEGG_04012_ErbB_signaling_pathway
CAMK2B	KEGG_04020_Calcium_signaling_pathway
CAMK2B	KEGG_04114_Oocyte_meiosis
CAMK2B	KEGG_04310_Wnt_signaling_pathway
CAMK2B	KEGG_04720_Long-term_potentiation
CAMK2B	KEGG_04722_Neurotrophin_signaling_pathway
CAMK2B	KEGG_04740_Olfactory_transduction
CAMK2B	KEGG_04912_GnRH_signaling_pathway
CAMK2B	KEGG_04916_Melanogenesis
CAMK2B	KEGG_05214_Glioma
MYLK	KEGG_04020_Calcium_signaling_pathway
MYLK	KEGG_04510_Focal_adhesion
MYLK	KEGG_04810_Regulation_of_actin_cytoskeleton
DRD4	KEGG_04080_Neuroactive_ligand-receptor_interaction
ST6GAL1	KEGG_00510_N-Glycan_biosynthesis
TYR	KEGG_00350_Tyrosine_metabolism
TYR	KEGG_00740_Riboflavin_metabolism
TYR	KEGG_00950_Isoquinoline_alkaloid_biosynthesis
TYR	KEGG_04916_Melanogenesis
GSK3B	KEGG_04012_ErbB_signaling_pathway
GSK3B	KEGG_04062_Chemokine_signaling_pathway
GSK3B	KEGG_04110_Cell_cycle
GSK3B	KEGG_04310_Wnt_signaling_pathway
GSK3B	KEGG_04340_Hedgehog_signaling_pathway
GSK3B	KEGG_04360_Axon_guidance
GSK3B	KEGG_04510_Focal_adhesion
GSK3B	KEGG_04660_T_cell_receptor_signaling_pathway
GSK3B	KEGG_04662_B_cell_receptor_signaling_pathway
GSK3B	KEGG_04722_Neurotrophin_signaling_pathway
GSK3B	KEGG_04910_Insulin_signaling_pathway
GSK3B	KEGG_04916_Melanogenesis
GSK3B	KEGG_05010_Alzheimer's_disease
GSK3B	KEGG_05200_Pathways_in_cancer

GSK3B	KEGG_05210_Colorectal_cancer
GSK3B	KEGG_05213_Endometrial_cancer
GSK3B	KEGG_05215_Prostate_cancer
GSK3B	KEGG_05217_Basal_cell_carcinoma
ABCB1	KEGG_02010_ABC_transporters
AKR1B1	KEGG_00040_Pentose_and_glucuronate_interconversions
AKR1B1	KEGG_00051_Fructose_and_mannose_metabolism
AKR1B1	KEGG_00052_Galactose_metabolism
AKR1B1	KEGG_00561_Glycerolipid_metabolism
AKR1B1	KEGG_00620_Pyruvate_metabolism
HSD17B2	KEGG_00140_Steroid_hormone_biosynthesis
ACHE	KEGG_00564_Glycerophospholipid_metabolism
MMP2	KEGG_04670_Leukocyte_transendothelial_migration
MMP2	KEGG_04912_GnRH_signaling_pathway
MMP2	KEGG_05200_Pathways_in_cancer
MMP2	KEGG_05219_Bladder_cancer
PYGL	KEGG_00500_Starch_and_sucrose_metabolism
PYGL	KEGG_04910_Insulin_signaling_pathway
MMP9	KEGG_04670_Leukocyte_transendothelial_migration
MMP9	KEGG_05200_Pathways_in_cancer
MMP9	KEGG_05219_Bladder_cancer
CSNK2A1	KEGG_04310_Wnt_signaling_pathway
CSNK2A1	KEGG_04520_Adherens_junction
CSNK2A1	KEGG_04530_Tight_junction
ABCC1	KEGG_02010_ABC_transporters
CA2	KEGG_00910_Nitrogen_metabolism
CDK6	KEGG_04110_Cell_cycle
CDK6	KEGG_04115_p53_signaling_pathway
CDK6	KEGG_05212_Pancreatic_cancer
CDK6	KEGG_05214_Glioma
CDK6	KEGG_05218_Melanoma
CDK6	KEGG_05220_Chronic_myeloid_leukemia
CDK6	KEGG_05222_Small_cell_lung_cancer
CDK6	KEGG_05223_Non-small_cell_lung_cancer
PIK3CG	KEGG_00562_Inositol_phosphate_metabolism
PIK3CG	KEGG_04012_ErbB_signaling_pathway
PIK3CG	KEGG_04062_Chemokine_signaling_pathway
PIK3CG	KEGG_04070_Phosphatidylinositol_signaling_system
PIK3CG	KEGG_04150_mTOR_signaling_pathway

PIK3CG	KEGG_04210_Apoptosis
PIK3CG	KEGG_04370_VEGF_signaling_pathway
PIK3CG	KEGG_04510_Focal_adhesion
PIK3CG	KEGG_04620_Toll-like_receptor_signaling_pathway
PIK3CG	KEGG_04630_Jak-STAT_signaling_pathway
PIK3CG	KEGG_04650_Natural_killer_cell_mediated_cytotoxicity
PIK3CG	KEGG_04660_T_cell_receptor_signaling_pathway
PIK3CG	KEGG_04662_B_cell_receptor_signaling_pathway
PIK3CG	KEGG_04664_Fc_epsilon_RI_signaling_pathway
PIK3CG	KEGG_04666_Fc_gamma_R-mediated_phagocytosis
PIK3CG	KEGG_04670_Leukocyte_transendothelial_migration
PIK3CG	KEGG_04722_Neurotrophin_signaling_pathway
PIK3CG	KEGG_04810_Regulation_of_actin_cytoskeleton
PIK3CG	KEGG_04910_Insulin_signaling_pathway
PIK3CG	KEGG_04914_Progesterone-mediated_oocyte_maturation
PIK3CG	KEGG_04930_Type_II_diabetes_mellitus
PIK3CG	KEGG_04960_Aldosterone-regulated_sodium_reabsorption
PIK3CG	KEGG_05100_Bacterial_invasion_of_epithelial_cells
PIK3CG	KEGG_05142_Chagas_disease
PIK3CG	KEGG_05200_Pathways_in_cancer
PIK3CG	KEGG_05210_Colorectal_cancer
PIK3CG	KEGG_05211_Renal_cell_carcinoma
PIK3CG	KEGG_05212_Pancreatic_cancer
PIK3CG	KEGG_05213_Endometrial_cancer
PIK3CG	KEGG_05214_Glioma
PIK3CG	KEGG_05215_Prostate_cancer
PIK3CG	KEGG_05218_Melanoma
PIK3CG	KEGG_05220_Chronic_myeloid_leukemia
PIK3CG	KEGG_05221_Acute_myeloid_leukemia
PIK3CG	KEGG_05222_Small_cell_lung_cancer
PIK3CG	KEGG_05223_Non-small_cell_lung_cancer
MET	KEGG_04060_Cytokine-cytokine_receptor_interaction
MET	KEGG_04144_Endocytosis
MET	KEGG_04360_Axon_guidance
MET	KEGG_04510_Focal_adhesion
MET	KEGG_04520_Adherens_junction
MET	KEGG_05100_Bacterial_invasion_of_epithelial_cells
MET	KEGG_05120_Epithelial_cell_signaling_in_Helicobacter_pylori_infection
MET	KEGG_05200_Pathways_in_cancer

MET	KEGG_05210_Colorectal_cancer
MET	KEGG_05211_Renal_cell_carcinoma
MET	KEGG_05218_Melanoma
CA9	KEGG_00910_Nitrogen_metabolism
HSD17B1	KEGG_00140_Steroid_hormone_biosynthesis
ALOX12	KEGG_00590_Arachidonic_acid_metabolism
AKR1A1	KEGG_00010_Glycolysis_/_Gluconeogenesis
AKR1A1	KEGG_00561_Glycerolipid_metabolism
AKR1A1	KEGG_00930_Caprolactam_degradation
ARG1	KEGG_00330_Arginine_and_proline_metabolism
ABCG2	KEGG_02010_ABC_transporters
ADORA3	KEGG_04080_Neuroactive_ligand-receptor_interaction
FLT3	KEGG_04060_Cytokine-cytokine_receptor_interaction
FLT3	KEGG_04640_Hematopoietic_cell_lineage
FLT3	KEGG_05200_Pathways_in_cancer
FLT3	KEGG_05221_Acute_myeloid_leukemia
CDK2	KEGG_04110_Cell_cycle
CDK2	KEGG_04114_Oocyte_meiosis
CDK2	KEGG_04115_p53_signaling_pathway
CDK2	KEGG_04914_Progesterone-mediated_oocyte_maturation
CDK2	KEGG_05215_Prostate_cancer
CDK2	KEGG_05222_Small_cell_lung_cancer
GLO1	KEGG_00620_Pyruvate_metabolism
AVPR2	KEGG_04080_Neuroactive_ligand-receptor_interaction
AVPR2	KEGG_04962_Vasopressin-regulated_water_reabsorption
KDR	KEGG_04060_Cytokine-cytokine_receptor_interaction
KDR	KEGG_04144_Endocytosis
KDR	KEGG_04370_VEGF_signaling_pathway
KDR	KEGG_04510_Focal_adhesion
ADORA2A	KEGG_04020_Calcium_signaling_pathway
ADORA2A	KEGG_04080_Neuroactive_ligand-receptor_interaction
ADORA2A	KEGG_04270_Vascular_smooth_muscle_contraction
CA6	KEGG_00910_Nitrogen_metabolism
CA1	KEGG_00910_Nitrogen_metabolism
CCNB1	KEGG_04110_Cell_cycle
CCNB1	KEGG_04115_p53_signaling_pathway
CCNB1	KEGG_04914_Progesterone-mediated_oocyte_maturation
PIM1	KEGG_04630_Jak-STAT_signaling_pathway
PIM1	KEGG_05221_Acute_myeloid_leukemia

CYP19A1	KEGG_00140_Steroid_hormone_biosynthesis
CYP1B1	KEGG_00140_Steroid_hormone_biosynthesis
CYP1B1	KEGG_00380_Tryptophan_metabolism
CYP1B1	KEGG_00980_Metabolism_of_xenobiotics_by_cytochrome_P450
IGF1R	KEGG_04114_Oocyte_meiosis
IGF1R	KEGG_04144_Endocytosis
IGF1R	KEGG_04510_Focal_adhesion
IGF1R	KEGG_04520_Adherens_junction
IGF1R	KEGG_04730_Long-term_depression
IGF1R	KEGG_04914_Progesterone-mediated_oocyte_maturation
IGF1R	KEGG_05200_Pathways_in_cancer
IGF1R	KEGG_05210_Colorectal_cancer
IGF1R	KEGG_05214_Glioma
IGF1R	KEGG_05215_Prostate_cancer
IGF1R	KEGG_05218_Melanoma
APP	KEGG_05010_Alzheimer's_disease
AKT1	KEGG_04010_MAPK_signaling_pathway
AKT1	KEGG_04012_ErbB_signaling_pathway
AKT1	KEGG_04062_Chemokine_signaling_pathway
AKT1	KEGG_04150_mTOR_signaling_pathway
AKT1	KEGG_04210_Apoptosis
AKT1	KEGG_04370_VEGF_signaling_pathway
AKT1	KEGG_04510_Focal_adhesion
AKT1	KEGG_04530_Tight_junction
AKT1	KEGG_04620_Toll-like_receptor_signaling_pathway
AKT1	KEGG_04630_Jak-STAT_signaling_pathway
AKT1	KEGG_04660_T_cell_receptor_signaling_pathway
AKT1	KEGG_04662_B_cell_receptor_signaling_pathway
AKT1	KEGG_04664_Fc_epsilon_RI_signaling_pathway
AKT1	KEGG_04666_Fc_gamma_R-mediated_phagocytosis
AKT1	KEGG_04722_Neurotrophin_signaling_pathway
AKT1	KEGG_04910_Insulin_signaling_pathway
AKT1	KEGG_04914_Progesterone-mediated_oocyte_maturation
AKT1	KEGG_04920_Adipocytokine_signaling_pathway
AKT1	KEGG_05142_Chagas_disease
AKT1	KEGG_05200_Pathways_in_cancer
AKT1	KEGG_05210_Colorectal_cancer
AKT1	KEGG_05211_Renal_cell_carcinoma
AKT1	KEGG_05212_Pancreatic_cancer

AKT1	KEGG_05213_Endometrial_cancer
AKT1	KEGG_05214_Glioma
AKT1	KEGG_05215_Prostate_cancer
AKT1	KEGG_05218_Melanoma
AKT1	KEGG_05220_Chronic_myeloid_leukemia
AKT1	KEGG_05221_Acute_myeloid_leukemia
AKT1	KEGG_05222_Small_cell_lung_cancer
AKT1	KEGG_05223_Non-small_cell_lung_cancer
PIK3R1	KEGG_04012_ErbB_signaling_pathway
PIK3R1	KEGG_04062_Chemokine_signaling_pathway
PIK3R1	KEGG_04070_Phosphatidylinositol_signaling_system
PIK3R1	KEGG_04150_mTOR_signaling_pathway
PIK3R1	KEGG_04210_Apoptosis
PIK3R1	KEGG_04370_VEGF_signaling_pathway
PIK3R1	KEGG_04510_Focal_adhesion
PIK3R1	KEGG_04620_Toll-like_receptor_signaling_pathway
PIK3R1	KEGG_04630_Jak-STAT_signaling_pathway
PIK3R1	KEGG_04650_Natural_killer_cell_mediated_cytotoxicity
PIK3R1	KEGG_04660_T_cell_receptor_signaling_pathway
PIK3R1	KEGG_04662_B_cell_receptor_signaling_pathway
PIK3R1	KEGG_04664_Fc_epsilon_RI_signaling_pathway
PIK3R1	KEGG_04666_Fc_gamma_R-mediated_phagocytosis
PIK3R1	KEGG_04670_Leukocyte_transendothelial_migration
PIK3R1	KEGG_04722_Neurotrophin_signaling_pathway
PIK3R1	KEGG_04810_Regulation_of_actin_cytoskeleton
PIK3R1	KEGG_04910_Insulin_signaling_pathway
PIK3R1	KEGG_04914_Progesterone-mediated_oocyte_maturation
PIK3R1	KEGG_04930_Type_II_diabetes_mellitus
PIK3R1	KEGG_04960_Aldosterone-regulated_sodium_reabsorption
PIK3R1	KEGG_05100_Bacterial_invasion_of_epithelial_cells
PIK3R1	KEGG_05142_Chagas_disease
PIK3R1	KEGG_05200_Pathways_in_cancer
PIK3R1	KEGG_05210_Colorectal_cancer
PIK3R1	KEGG_05211_Renal_cell_carcinoma
PIK3R1	KEGG_05212_Pancreatic_cancer
PIK3R1	KEGG_05213_Endometrial_cancer
PIK3R1	KEGG_05214_Glioma
PIK3R1	KEGG_05215_Prostate_cancer
PIK3R1	KEGG_05218_Melanoma

PIK3R1	KEGG_05220_Chronic_myeloid_leukemia
PIK3R1	KEGG_05221_Acute_myeloid_leukemia
PIK3R1	KEGG_05222_Small_cell_lung_cancer
PIK3R1	KEGG_05223_Non-small_cell_lung_cancer
EGFR	KEGG_04010_MAPK_signaling_pathway
EGFR	KEGG_04012_ErbB_signaling_pathway
EGFR	KEGG_04020_Calcium_signaling_pathway
EGFR	KEGG_04060_Cytokine-cytokine_receptor_interaction
EGFR	KEGG_04144_Endocytosis
EGFR	KEGG_04320_Dorso-ventral_axis_formation
EGFR	KEGG_04510_Focal_adhesion
EGFR	KEGG_04520_Adherens_junction
EGFR	KEGG_04540_Gap_junction
EGFR	KEGG_04810_Regulation_of_actin_cytoskeleton
EGFR	KEGG_04912_GnRH_signaling_pathway
EGFR	KEGG_05120_Epithelial_cell_signaling_in_Helicobacter_pylori_infection
EGFR	KEGG_05200_Pathways_in_cancer
EGFR	KEGG_05210_Colorectal_cancer
EGFR	KEGG_05212_Pancreatic_cancer
EGFR	KEGG_05213_Endometrial_cancer
EGFR	KEGG_05214_Glioma
EGFR	KEGG_05215_Prostate_cancer
EGFR	KEGG_05218_Melanoma
EGFR	KEGG_05219_Bladder_cancer
EGFR	KEGG_05223_Non-small_cell_lung_cancer
CCNB3	KEGG_04110_Cell_cycle
CCNB3	KEGG_04115_p53_signaling_pathway
CCNB3	KEGG_04914_Progesterone-mediated_oocyte_maturation
AKR1C2	KEGG_00980_Metabolism_of_xenobiotics_by_cytochrome_P450
CCNB2	KEGG_04110_Cell_cycle
CCNB2	KEGG_04115_p53_signaling_pathway
CCNB2	KEGG_04914_Progesterone-mediated_oocyte_maturation
XDH	KEGG_00230_Purine_metabolism
XDH	KEGG_00232_Caffeine_metabolism
XDH	KEGG_00983_Drug_metabolism_-_other_enzymes
ALOX15	KEGG_00590_Arachidonic_acid_metabolism
ALOX15	KEGG_00591_Linoleic_acid_metabolism
ADORA1	KEGG_04080_Neuroactive_ligand-receptor_interaction
CA3	KEGG_00910_Nitrogen_metabolism

CDK5	KEGG_04360_Axon_guidance
CDK5	KEGG_05010_Alzheimer's_disease
SYK	KEGG_04650_Natural_killer_cell_mediated_cytotoxicity
SYK	KEGG_04662_B_cell_receptor_signaling_pathway
SYK	KEGG_04664_Fc_epsilon_RI_signaling_pathway
SYK	KEGG_04666_Fc_gamma_R-mediated_phagocytosis
PLK1	KEGG_04110_Cell_cycle
PLK1	KEGG_04114_Oocyte_meiosis
PLK1	KEGG_04914_Progesterone-mediated_oocyte_maturation
CA4	KEGG_00910_Nitrogen_metabolism
CA7	KEGG_00910_Nitrogen_metabolism
PTK2	KEGG_04012_ErbB_signaling_pathway
PTK2	KEGG_04062_Chemokine_signaling_pathway
PTK2	KEGG_04360_Axon_guidance
PTK2	KEGG_04370_VEGF_signaling_pathway
PTK2	KEGG_04510_Focal_adhesion
PTK2	KEGG_04670_Leukocyte_transendothelial_migration
PTK2	KEGG_04810_Regulation_of_actin_cytoskeleton
PTK2	KEGG_05100_Bacterial_invasion_of_epithelial_cells
PTK2	KEGG_05200_Pathways_in_cancer
PTK2	KEGG_05222_Small_cell_lung_cancer
PLA2G1B	KEGG_00564_Glycerophospholipid_metabolism
PLA2G1B	KEGG_00565_Ether_lipid_metabolism
PLA2G1B	KEGG_00590_Arachidonic_acid_metabolism
PLA2G1B	KEGG_00591_Linoleic_acid_metabolism
PLA2G1B	KEGG_00592_alpha-Linolenic_acid_metabolism
PLA2G1B	KEGG_04010_MAPK_signaling_pathway
PLA2G1B	KEGG_04270_Vascular_smooth_muscle_contraction
PLA2G1B	KEGG_04370_VEGF_signaling_pathway
PLA2G1B	KEGG_04664_Fc_epsilon_RI_signaling_pathway
PLA2G1B	KEGG_04730_Long-term_depression
PLA2G1B	KEGG_04912_GnRH_signaling_pathway
INSR	KEGG_04520_Adherens_junction
INSR	KEGG_04910_Insulin_signaling_pathway
INSR	KEGG_04930_Type_II_diabetes_mellitus
INSR	KEGG_04960_Aldosterone-regulated_sodium_reabsorption
CA5A	KEGG_00910_Nitrogen_metabolism
CDK5R1	KEGG_05010_Alzheimer's_disease
GPR35	KEGG_04080_Neuroactive_ligand-receptor_interaction

F2	KEGG_04080_Neuroactive_ligand-receptor_interaction
F2	KEGG_04610_Complement_and_coagulation_cascades
F2	KEGG_04810_Regulation_of_actin_cytoskeleton
BACE1	KEGG_05010_Alzheimer's_disease
MAPT	KEGG_04010_MAPK_signaling_pathway
MAPT	KEGG_05010_Alzheimer's_disease
AKR1C1	KEGG_00980_Metabolism_of_xenobiotics_by_cytochrome_P450
MAOA	KEGG_00260_Glycine__serine_and_threonine_metabolism
MAOA	KEGG_00330_Arginine_and_proline_metabolism
MAOA	KEGG_00340_Histidine_metabolism
MAOA	KEGG_00350_Tyrosine_metabolism
MAOA	KEGG_00360_Phenylalanine_metabolism
MAOA	KEGG_00380_Tryptophan_metabolism
MAOA	KEGG_00982_Drug_metabolism_-_cytochrome_P450
AKR1C3	KEGG_00590_Arachidonic_acid_metabolism
AKR1C3	KEGG_00980_Metabolism_of_xenobiotics_by_cytochrome_P450
DAPK1	KEGG_05200_Pathways_in_cancer
DAPK1	KEGG_05219_Bladder_cancer
SRC	KEGG_04012_ErbB_signaling_pathway
SRC	KEGG_04370_VEGF_signaling_pathway
SRC	KEGG_04510_Focal_adhesion
SRC	KEGG_04520_Adherens_junction
SRC	KEGG_04530_Tight_junction
SRC	KEGG_04540_Gap_junction
SRC	KEGG_04912_GnRH_signaling_pathway
SRC	KEGG_05100_Bacterial_invasion_of_epithelial_cells
SRC	KEGG_05120_Epithelial_cell_signaling_in_Helicobacter_pylori_infection
AKR1C4	KEGG_00120_Primary_bile_acid_biosynthesis
AKR1C4	KEGG_00140_Steroid_hormone_biosynthesis
AKR1C4	KEGG_00980_Metabolism_of_xenobiotics_by_cytochrome_P450
GSK3B	KEGG_04392_Hippo_Signaling_Pathway
MPG	KEGG_03410_Base_excision_repair
APEX1	KEGG_03410_Base_excision_repair
EGFR	KEGG_04014_Ras_signaling_pathway_-_Homo_sapiens_(human)
AKT1	KEGG_04014_Ras_signaling_pathway_-_Homo_sapiens_(human)
IGF1R	KEGG_04014_Ras_signaling_pathway_-_Homo_sapiens_(human)
INSR	KEGG_04014_Ras_signaling_pathway_-_Homo_sapiens_(human)
KDR	KEGG_04014_Ras_signaling_pathway_-_Homo_sapiens_(human)
MET	KEGG_04014_Ras_signaling_pathway_-_Homo_sapiens_(human)

PIK3R1	KEGG_04014_Ras_signaling_pathway_-_Homo_sapiens_(human)
PLA2G1B	KEGG_04014_Ras_signaling_pathway_-_Homo_sapiens_(human)
ADORA2A	KEGG_04015_Rap1_signaling_pathway_-_Homo_sapiens_(human)
EGFR	KEGG_04015_Rap1_signaling_pathway_-_Homo_sapiens_(human)
AKT1	KEGG_04015_Rap1_signaling_pathway_-_Homo_sapiens_(human)
IGF1R	KEGG_04015_Rap1_signaling_pathway_-_Homo_sapiens_(human)
INSR	KEGG_04015_Rap1_signaling_pathway_-_Homo_sapiens_(human)
KDR	KEGG_04015_Rap1_signaling_pathway_-_Homo_sapiens_(human)
MET	KEGG_04015_Rap1_signaling_pathway_-_Homo_sapiens_(human)
PIK3R1	KEGG_04015_Rap1_signaling_pathway_-_Homo_sapiens_(human)
SRC	KEGG_04015_Rap1_signaling_pathway_-_Homo_sapiens_(human)
AKT1	KEGG_04371_Apelin_signaling_pathway_-_Homo_sapiens_(human)
MYLK	KEGG_04371_Apelin_signaling_pathway_-_Homo_sapiens_(human)
PIK3CG	KEGG_04371_Apelin_signaling_pathway_-_Homo_sapiens_(human)
PARP1	KEGG_04064_NF-kappa_B_signaling_pathway_-_Homo_sapiens_(human)
CSNK2A1	KEGG_04064_NF-kappa_B_signaling_pathway_-_Homo_sapiens_(human)
SYK	KEGG_04064_NF-kappa_B_signaling_pathway_-_Homo_sapiens_(human)
AKT1	KEGG_04668_TNF_signaling_pathway_-_Homo_sapiens_(human)
MMP3	KEGG_04668_TNF_signaling_pathway_-_Homo_sapiens_(human)
MMP9	KEGG_04668_TNF_signaling_pathway_-_Homo_sapiens_(human)
PIK3R1	KEGG_04668_TNF_signaling_pathway_-_Homo_sapiens_(human)
EGFR	KEGG_04066_HIF-1_signaling_pathway_-_Homo_sapiens_(human)
AKT1	KEGG_04066_HIF-1_signaling_pathway_-_Homo_sapiens_(human)
IGF1R	KEGG_04066_HIF-1_signaling_pathway_-_Homo_sapiens_(human)
INSR	KEGG_04066_HIF-1_signaling_pathway_-_Homo_sapiens_(human)
PIK3R1	KEGG_04066_HIF-1_signaling_pathway_-_Homo_sapiens_(human)
CAMK2B	KEGG_04066_HIF-1_signaling_pathway_-_Homo_sapiens_(human)
CDK2	KEGG_04068_FoxO_signaling_pathway_-_Homo_sapiens_(human)
EGFR	KEGG_04068_FoxO_signaling_pathway_-_Homo_sapiens_(human)
AKT1	KEGG_04068_FoxO_signaling_pathway_-_Homo_sapiens_(human)
IGF1R	KEGG_04068_FoxO_signaling_pathway_-_Homo_sapiens_(human)
INSR	KEGG_04068_FoxO_signaling_pathway_-_Homo_sapiens_(human)
PIK3R1	KEGG_04068_FoxO_signaling_pathway_-_Homo_sapiens_(human)
PLK1	KEGG_04068_FoxO_signaling_pathway_-_Homo_sapiens_(human)
CCNB3	KEGG_04068_FoxO_signaling_pathway_-_Homo_sapiens_(human)
CCNB1	KEGG_04068_FoxO_signaling_pathway_-_Homo_sapiens_(human)
CCNB2	KEGG_04068_FoxO_signaling_pathway_-_Homo_sapiens_(human)
EGFR	KEGG_04072_Phospholipase_D_signaling_pathway_-_Homo_sapiens_(human)

AKT1	KEGG_04072_Phospholipase_D_signaling_pathway_-_Homo_sapiens_(human)
CXCR1	KEGG_04072_Phospholipase_D_signaling_pathway_-_Homo_sapiens_(human)
INSR	KEGG_04072_Phospholipase_D_signaling_pathway_-_Homo_sapiens_(human)
PIK3CG	KEGG_04072_Phospholipase_D_signaling_pathway_-_Homo_sapiens_(human)
PIK3R1	KEGG_04072_Phospholipase_D_signaling_pathway_-_Homo_sapiens_(human)
AVPR2	KEGG_04072_Phospholipase_D_signaling_pathway_-_Homo_sapiens_(human)
SYK	KEGG_04072_Phospholipase_D_signaling_pathway_-_Homo_sapiens_(human)
ADORA1	KEGG_04071_Sphingolipid_signaling_pathway_-_Homo_sapiens_(human)
ADORA3	KEGG_04071_Sphingolipid_signaling_pathway_-_Homo_sapiens_(human)
AKT1	KEGG_04071_Sphingolipid_signaling_pathway_-_Homo_sapiens_(human)
ABCC1	KEGG_04071_Sphingolipid_signaling_pathway_-_Homo_sapiens_(human)
ABCC1	KEGG_04071_Sphingolipid_signaling_pathway_-_Homo_sapiens_(human)
PIK3R1	KEGG_04071_Sphingolipid_signaling_pathway_-_Homo_sapiens_(human)
ADORA1	KEGG_04024_cAMP_signaling_pathway_-_Homo_sapiens_(human)
ADORA2A	KEGG_04024_cAMP_signaling_pathway_-_Homo_sapiens_(human)
AKT1	KEGG_04024_cAMP_signaling_pathway_-_Homo_sapiens_(human)
PIK3R1	KEGG_04024_cAMP_signaling_pathway_-_Homo_sapiens_(human)
CAMK2B	KEGG_04024_cAMP_signaling_pathway_-_Homo_sapiens_(human)
ADORA1	KEGG_04022_cGMP-PKG_signaling_pathway_-_Homo_sapiens_(human)
ADORA3	KEGG_04022_cGMP-PKG_signaling_pathway_-_Homo_sapiens_(human)
AKT1	KEGG_04022_cGMP-PKG_signaling_pathway_-_Homo_sapiens_(human)
INSR	KEGG_04022_cGMP-PKG_signaling_pathway_-_Homo_sapiens_(human)
MYLK	KEGG_04022_cGMP-PKG_signaling_pathway_-_Homo_sapiens_(human)
PIK3CG	KEGG_04022_cGMP-PKG_signaling_pathway_-_Homo_sapiens_(human)
CDK2	KEGG_04151_PI3K-Akt_signaling_pathway_-_Homo_sapiens_(human)
CDK6	KEGG_04151_PI3K-Akt_signaling_pathway_-_Homo_sapiens_(human)
EGFR	KEGG_04151_PI3K-Akt_signaling_pathway_-_Homo_sapiens_(human)
AKT1	KEGG_04151_PI3K-Akt_signaling_pathway_-_Homo_sapiens_(human)
GSK3B	KEGG_04151_PI3K-Akt_signaling_pathway_-_Homo_sapiens_(human)
IGF1R	KEGG_04151_PI3K-Akt_signaling_pathway_-_Homo_sapiens_(human)
INSR	KEGG_04151_PI3K-Akt_signaling_pathway_-_Homo_sapiens_(human)
KDR	KEGG_04151_PI3K-Akt_signaling_pathway_-_Homo_sapiens_(human)
MET	KEGG_04151_PI3K-Akt_signaling_pathway_-_Homo_sapiens_(human)
PIK3CG	KEGG_04151_PI3K-Akt_signaling_pathway_-_Homo_sapiens_(human)

PIK3R1	KEGG_04151_PI3K-Akt_signaling_pathway_-_Homo_sapiens_(human)
PKN1	KEGG_04151_PI3K-Akt_signaling_pathway_-_Homo_sapiens_(human)
PTK2	KEGG_04151_PI3K-Akt_signaling_pathway_-_Homo_sapiens_(human)
SYK	KEGG_04151_PI3K-Akt_signaling_pathway_-_Homo_sapiens_(human)
AKT1	KEGG_04152_AMPK_signaling_pathway_-_Homo_sapiens_(human)
IGF1R	KEGG_04152_AMPK_signaling_pathway_-_Homo_sapiens_(human)
INSR	KEGG_04152_AMPK_signaling_pathway_-_Homo_sapiens_(human)
PIK3R1	KEGG_04152_AMPK_signaling_pathway_-_Homo_sapiens_(human)
MPO	KEGG_04145_Phagosome
XDH	KEGG_04146_Peroxisome
AKT1	KEGG_04550_Signaling_pathways_regulating_pluripotency_of_stem_cells
GSK3B	KEGG_04550_Signaling_pathways_regulating_pluripotency_of_stem_cells
IGF1R	KEGG_04550_Signaling_pathways_regulating_pluripotency_of_stem_cells
PIK3R1	KEGG_04550_Signaling_pathways_regulating_pluripotency_of_stem_cells
ALOX12	KEGG_04750_Inflammatory_mediator_regulation_of_TRP_channels
PIK3R1	KEGG_04750_Inflammatory_mediator_regulation_of_TRP_channels
SRC	KEGG_04750_Inflammatory_mediator_regulation_of_TRP_channels
CAMK2B	KEGG_04750_Inflammatory_mediator_regulation_of_TRP_channels
AKT1	KEGG_04380_Osteoclast_differentiation
PIK3R1	KEGG_04380_Osteoclast_differentiation
SYK	KEGG_04380_Osteoclast_differentiation
CAMK2B	KEGG_04713_Circadian_entrainment
AKT1	KEGG_04211_Longevity_regulating_pathway
IGF1R	KEGG_04211_Longevity_regulating_pathway
INSR	KEGG_04211_Longevity_regulating_pathway
PIK3R1	KEGG_04211_Longevity_regulating_pathway
FLT3	KEGG_04640_Hematopoietic_cell_lineage
CD38	KEGG_04640_Hematopoietic_cell_lineage
AKT1	KEGG_04611_Platelet_activation
MYLK	KEGG_04611_Platelet_activation
PIK3CG	KEGG_04611_Platelet_activation
PIK3R1	KEGG_04611_Platelet_activation
SRC	KEGG_04611_Platelet_activation
SYK	KEGG_04611_Platelet_activation
CYP1B1	KEGG_04913_Ovarian_steroidogenesis
CYP19A1	KEGG_04913_Ovarian_steroidogenesis
ALOX5	KEGG_04913_Ovarian_steroidogenesis
ALOX5	KEGG_04913_Ovarian_steroidogenesis
HSD17B1	KEGG_04913_Ovarian_steroidogenesis

HSD17B2	KEGG_04913_Ovarian_steroidogenesis
IGF1R	KEGG_04913_Ovarian_steroidogenesis
INSR	KEGG_04913_Ovarian_steroidogenesis
AKR1C3	KEGG_04913_Ovarian_steroidogenesis
EGFR	KEGG_04915_Estrogen_signaling_pathway
AKT1	KEGG_04915_Estrogen_signaling_pathway
ESR2	KEGG_04915_Estrogen_signaling_pathway
MMP2	KEGG_04915_Estrogen_signaling_pathway
MMP9	KEGG_04915_Estrogen_signaling_pathway
PIK3R1	KEGG_04915_Estrogen_signaling_pathway
SRC	KEGG_04915_Estrogen_signaling_pathway
AKT1	KEGG_04917_Prolactin_signaling_pathway
ESR2	KEGG_04917_Prolactin_signaling_pathway
GSK3B	KEGG_04917_Prolactin_signaling_pathway
PIK3R1	KEGG_04917_Prolactin_signaling_pathway
SRC	KEGG_04917_Prolactin_signaling_pathway
EGFR	KEGG_04921_Oxytocin_signaling_pathway
MYLK	KEGG_04921_Oxytocin_signaling_pathway
PIK3CG	KEGG_04921_Oxytocin_signaling_pathway
SRC	KEGG_04921_Oxytocin_signaling_pathway
CAMK2B	KEGG_04921_Oxytocin_signaling_pathway
CD38	KEGG_04921_Oxytocin_signaling_pathway
TTR	KEGG_04918_Thyroid_hormone_synthesis
AKT1	KEGG_04919_Thyroid_hormone_signaling_pathway
GSK3B	KEGG_04919_Thyroid_hormone_signaling_pathway
PIK3R1	KEGG_04919_Thyroid_hormone_signaling_pathway
SRC	KEGG_04919_Thyroid_hormone_signaling_pathway
ADORA1	KEGG_04924_Renin_secretion
AKT1	KEGG_04261_Adrenergic_signaling_in_cardiomyocytes
PIK3CG	KEGG_04261_Adrenergic_signaling_in_cardiomyocytes
CAMK2B	KEGG_04261_Adrenergic_signaling_in_cardiomyocytes
MMP2	KEGG_05418_Fluid_shear_stress_and_atherosclerosis
MMP9	KEGG_05418_Fluid_shear_stress_and_atherosclerosis
PIK3R1	KEGG_05418_Fluid_shear_stress_and_atherosclerosis
KDR	KEGG_05418_Fluid_shear_stress_and_atherosclerosis
SRC	KEGG_05418_Fluid_shear_stress_and_atherosclerosis
PTK2	KEGG_05418_Fluid_shear_stress_and_atherosclerosis
